# Unraveling NETs in Sepsis: From Cellular Mechanisms to Clinical Relevance

**DOI:** 10.3390/ijms26157464

**Published:** 2025-08-01

**Authors:** Giulia Pignataro, Stefania Gemma, Martina Petrucci, Fabiana Barone, Andrea Piccioni, Francesco Franceschi, Marcello Candelli

**Affiliations:** Department of Emergency, Anesthesiological and Reanimation Sciences, Fondazione Policlinico Universitario A. Gemelli, IRCCS, 00168 Rome, Italy; stefania.gemma@policliniocgemelli.it (S.G.); martina.petrucci@policlinicogemelli.it (M.P.); fabiana.barone@policlinicogemelli.it (F.B.); andrea.piccioni@policlinicogemelli.it (A.P.); francesco.franceschi@policlinicogemelli.it (F.F.)

**Keywords:** NETs, NETosis, sepsis, immunothrombosis, biomarkers, targeted therapy

## Abstract

Sepsis is a clinical syndrome characterized by a dysregulated host response to infection, frequently resulting in septic shock and multi-organ failure. Emerging evidence highlights the critical role of neutrophil extracellular traps (NETs) in the pathophysiology of sepsis. NETs are extracellular structures composed of chromatin DNA, histones, and granular proteins released by neutrophils through a specialized form of cell death known as NETosis. While NETs contribute to the containment of pathogens, their excessive or dysregulated production in sepsis is associated with endothelial damage, immunothrombosis, and organ dysfunction. Several NET-associated biomarkers have been identified, including circulating cell-free DNA (cfDNA), histones, MPO-DNA complexes, and neutrophil elastase–DNA complexes, which correlate with the disease severity and prognosis. Therapeutic strategies targeting NETs are currently under investigation. Inhibition of NET formation using PAD4 inhibitors or ROS scavengers has shown protective effects in preclinical models. Conversely, DNase I therapy facilitates the degradation of extracellular DNA, reducing the NET-related cytotoxicity and thrombotic potential. Additionally, heparin and its derivatives have demonstrated the ability to neutralize NET-associated histones and mitigate coagulopathy. Novel approaches include targeting upstream signaling pathways, such as TLR9 and IL-8/CXCR2, offering further therapeutic promise.

## 1. Introduction

Sepsis is characterized by the severe impairment of organ function due to an abnormal and uncontrolled immune response to infection. In clinical practice, this dysfunction can be identified through an increase of at least two points in the Sequential [Sepsis-Related] Organ Failure Assessment (SOFA) score, a change that correlates with an in-hospital mortality rate of over 10% [1]. It is a significant global health issue, substantially impacting healthcare systems in terms of both mortality and costs. Despite a decline in its incidence and mortality over the past decades, sepsis remained a major global health concern in 2017, with an estimated 48.9 million cases worldwide and 11 million associated deaths, accounting for approximately 19.7% of the total deaths, even if regional variations must be considered [2]. In the United States, the incidence of severe sepsis can reach up to 3 million cases, with in-hospital mortality rates as high as 29%, according to some statistics [3]. In sepsis, the host immune system mounts a response—of varying appropriateness—against molecules released either by the pathogen (pathogen-associated molecular patterns, PAMPs) or by the host itself (damage-associated molecular patterns, DAMPs). The extent of the resulting inflammatory response can, in turn, contribute to tissue injury and host damage [4]. Several inflammatory cells play a role in the pathogenesis of sepsis, with neutrophils being particularly prominent. These cells contribute to pathogen defense through various mechanisms, including the release of toxic substances such as proteolytic enzymes and reactive oxygen species (ROS), phagocytosis, and the formation of neutrophil extracellular traps (NETs) [5]. Neutrophils and NETs contribute to an immune system imbalance by inducing inflammatory and angiogenic responses in endothelial cells. This activation accelerates glycolysis, exacerbates oxidative stress, and damages the endothelial glycocalyx. Therefore, the endothelial barrier’s integrity is compromised due to junctional disruption, increased adhesion molecule expression, and programmed cell death. Additionally, NETs shift endothelial cells toward a procoagulant state by disrupting anticoagulant pathways and enhancing tissue factor expression. These changes lead to excessive vascular leakage, resulting in hypotension, tissue swelling, and ultimately, organ failure in sepsis [6,7]. In this review, we explore the role of NETs in sepsis, focusing on their contribution to immune dysregulation, endothelial dysfunction, and disease progression.

## 2. Neutrophil Extracellular Traps (NETs): Formation and Function

Neutrophils, the most abundant leukocytes in the bloodstream, serve as the body’s first line of defense against pathogens, playing a central role in innate immunity. These highly dynamic and complex cells interact with both immune and non-immune cells, extending their function beyond microbial killing. They are crucial for adaptive immune activation, wound healing, and inflammation resolution. Their defense mechanisms include phagocytosis, direct pathogen killing through the release of granule enzymes, ROS production, recruitment of other immune cells, and, finally, NETosis [8,9]. NETs are a component of innate immunity involved in the antimicrobial response. They consist of fibers composed of granule proteins, primarily azurophilic ones including neutrophil elastase (NE) and myeloperoxidase (MPO), and chromatin. They trap and kill Gram-positive and Gram-negative bacteria, as well as fungi, and degrade virulence factors, ensuring high concentrations of antimicrobials at the site of infection [10,11]. Having distinct functions other than phagocytosis, NETs appear to be produced and released in response to microorganisms for which phagocytosis is ineffective. This suggests the possibility that neutrophils possess sensors enabling them to select the most effective immune response to deploy [12].

### 2.1. NETosis

#### 2.1.1. Types of NETosis

Currently, studies mainly describe two types of NETosis: suicidal NETosis, in which the release of NETs is a consequence of neutrophil death, and vital NETosis, which occurs in the absence of cell death.

The former, i.e., suicidal NETosis, is the predominant mechanism [13]. In this case, the release of NETs results from a distinct form of cell death, separate from both apoptosis and necrosis. Neutrophils for which this pathway is activated undergo a series of morphological changes, beginning with the loss of differentiation between euchromatin and heterochromatin, followed by nuclear membrane separation, vesicular membrane dissolution, and the fragmentation of the nuclear envelope into vesicles. This process allows nuclear and cytoplasmic components to mix before NET release, which occurs upon plasma membrane rupture. This form of neutrophil death depends on ROS production by NADPH oxidase [14]. Specifically, NADPH-derived ROS promote the release of NE and MPO from azurophilic granules and their translocation to the nucleus. Notably, ROS facilitate the release of NE into the cytosol via an MPO-dependent mechanism. Once in the cytosol, NE interacts with and degrades F-actin, disrupting the cytoskeleton and allowing proteases to translocate into the nucleus. NE degrades histones, while MPO binds chromatin, further promoting chromatin decondensation [15,16]. In models of complete MPO deficiency, neutrophils fail to generate NETs, increasing the susceptibility to severe infections. Partial MPO inhibition delays and reduces NET formation, highlighting MPO’s role in this process [17]. Additionally, histone citrullination, mediated by peptidyl arginine deiminase 4 (PAD4), has been implicated in NETosis and studied in neutrophil-like human leukemia 60 (HL-60) cells. Beyond their antimicrobial function, NETs sequester toxic proteins to prevent excessive tissue damage. Their association with DNA modulates their degradation, balancing antimicrobial efficacy with host protection during sepsis [15]. Studies using PAD4 knockout (PAD4^−/−^) mice have further demonstrated that this enzyme is critical for NET formation. PAD4-deficient neutrophils fail to produce NETs in response to stimuli such as chemokines or bacteria. As a result, PAD4^−/−^ mice exhibit greater susceptibility to infections compared to controls due to the absence of NETs. Moreover, histone citrullination appears to reduce their direct antimicrobial activity, suggesting that PAD4 primarily facilitates chromatin decondensation for NET formation rather than enhancing histone-mediated bacterial killing [18].

Unlike the mechanism just described, vital NETosis is a process in which the release of NETs does not accompany neutrophil death but occurs while preserving the membrane’s integrity. Compared to suicidal NETosis, which is slower, this form is faster, taking between 5 and 60 min to complete after the stimulus, and is independent of ROS. Furthermore, although previous observations suggested that NETosis is a slow process that impairs neutrophil function, recent data indicate that neutrophils can have two distinct fates during NETosis. Some cells lose their nuclei entirely, while others retain diffuse nuclear material within the cytoplasm. Importantly, anuclear neutrophils preserve essential functions such as chemotaxis and phagocytosis [13,19].

A third distinct form of NETosis, first described in 2009, occurs when neutrophils, in response to specific stimuli such as the complement component C5a, release NETs composed of mitochondrial DNA (mtDNA) instead of nuclear DNA. This pathway appears to be ROS-dependent, similar to suicidal NETosis, but unlike that form, the neutrophils remain viable throughout the process [20].

#### 2.1.2. Stimuli for NETosis

Microbial infections are potent triggers of NETosis. Platelets appear to play a crucial role in neutrophil activation during these events. The interaction between platelets and neutrophils significantly enhances the antimicrobial response by promoting NET formation. Lipopolysaccharide (LPS) from Gram-negative bacteria, which alone is often insufficient to induce NET release by neutrophils, can activate toll-like receptor 4 (TLR4) expressed on the platelet surface. Platelet TLR4 activation promotes adhesion to neutrophils, leading to their activation and subsequent NET release. This platelet-mediated activation is particularly evident during severe systemic infections and takes place predominantly at the microvascular endothelium, where it can contribute to endothelial damage [21]. Carestia et al., investigating the role of platelets in neutrophil activity, demonstrated that, in response to specific stimuli such as LPS from Gram-negative bacteria and Pam3 cysteine–serine–lysine from Gram-positive bacteria, platelets are capable of inducing NETosis. Specifically, these stimuli trigger the production of thromboxane A_2_, which in turn promotes the release of von Willebrand factor and platelet factor 4. In the presence of platelet glycoprotein Ib and neutrophil cluster of differentiation 18 (CD18), these mediators facilitate the formation of neutrophil extracellular traps. Supporting this mechanism, aspirin, by inhibiting thromboxane A_2_ synthesis, was shown to block platelet-mediated NET formation [22].

Gram-positive bacteria such as Streptococcus and Staphylococcus species are also capable of inducing NET formation. One study demonstrated that the activation of neutrophils by Gram-positive bacteria requires the simultaneous engagement of toll-like receptor 2 (TLR2) and the complement component C3a receptor [19].

**Figure 1:**
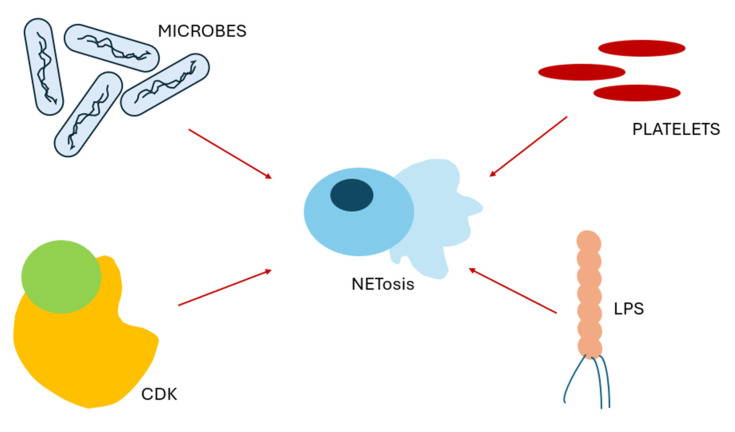
Stimuli for NETosis.

Other molecules that have been investigated in relation to NETosis include cyclin-dependent kinases (CDKs), which are key regulators of the cell cycle. Although neutrophils are terminally differentiated and non-proliferative cells, studies have shown that they can re-engage certain cell cycle signaling pathways during NET formation, particularly those involving CDK4/6. Inhibition of CDK activity has been found to decrease NET production, while neutrophils lacking p21—a natural CDK inhibitor—exhibit enhanced NET release and have been linked to autoimmune conditions such as lupus. CDK6, in particular, plays a critical role in neutrophil-mediated defense against Candida albicans, as mice deficient in CDK6 show increased susceptibility to fungal infections [23] (Figure 1).

#### 2.1.3. Influences of Neutrophil Phenotype on NETosis

A growing body of evidence highlights that neutrophil heterogeneity significantly shapes NET formation under both homeostatic and pathological conditions [24]. Specific phenotypic subsets, such as ICAM-1^+^ neutrophils, exhibit enhanced NETotic potential. This population appears to be expanded during sepsis and is stimulated by cold-inducible RNA-binding protein (CIRP), which promotes NETosis through iNOS upregulation, as shown in CIRP-treated bone marrow-derived neutrophils [25]. Functionally, ICAM-1 not only marks pro-NETotic neutrophils but also regulates their tissue infiltration. In murine models, a ICAM-1 blockade selectively impairs neutrophil accumulation in the lungs, spleen, and thymus while facilitating their migration to the peritoneal cavity, suggesting that ICAM-1 modulates compartment-specific inflammation [26].

Metabolic cues also contribute to NET regulation: d(−) lactic acid, a metabolite whose levels are elevated in certain inflammatory contexts, induces NETosis by promoting PAD4-dependent chromatin decondensation and requires transport through monocarboxylate transporter 1 (MCT1). These NETs are enriched in CD11b and citrullinated histone H4 and promote neutrophil–endothelial adhesion via CD11b/ICAM-1 interactions [27].

Density-based subsets of neutrophils also show differential NETotic capacities. Low-density neutrophils (LDNs), often associated with chronic inflammation and cancer, exhibit a more activated phenotype with elevated ROS and NET production compared to high-density neutrophils (HDNs) [28,29].

Moreover, microbial components modulate neutrophil aging through TLR–MyD88 signaling pathways. Aged neutrophils, characterized by increased CXCR4 and reduced L-selectin, display enhanced integrin αMβ2 activation and a pronounced ability to release NETs during inflammatory responses [30].

Together, these findings underscore the functional plasticity of neutrophils and highlight how phenotype-specific traits and environmental cues converge to regulate NET formation in health and disease.

## 3. Role of NETs in Sepsis

### 3.1. Beneficial Role of NETs in Sepsis

NETs play a fundamental role in the innate immune response, especially during severe infections like sepsis. These web-like structures, composed of DNA and antimicrobial proteins, can effectively trap and neutralize invading pathogens, acting as both a physical and chemical barrier. In the case of group A Streptococcus (GAS), NETs serve as a critical defense mechanism. GAS, however, has evolved strategies to counteract this response, such as the production of the DNase Sda1, which degrades NETs and enhances bacterial virulence. Inhibiting this enzyme restores the bactericidal capacity of neutrophils, confirming the protective role of NETs and pointing to potential therapeutic strategies that preserve their integrity [31].

During sepsis, NETs are released directly into the liver sinusoids, where they help prevent systemic dissemination of pathogens. This localization is particularly strategic, as the liver filters both systemic blood and intestinal drainage, making it a frontline organ in pathogen containment. The formation of NETs in this context is dependent on direct interactions between neutrophils and activated platelets, underscoring the immunological role of platelets. While this mechanism enhances microbial clearance, it can also contribute to tissue injury via coagulation activation and the cytotoxic effects of NET components like histones. Indeed, experimental removal of NETs in septic mice reduces liver damage but compromises infection control, highlighting the need for a balanced therapeutic approach [32].

Some pathogens, like Streptococcus pneumoniae, evade NET-mediated killing despite being trapped within NETs. This entrapment still plays a valuable role by containing the infection within the lungs and limiting its systemic spread. Pneumococci can express surface nucleases like EndA that degrade the DNA backbone of NETs, facilitating bacterial escape and dissemination from the respiratory tract to the bloodstream. In addition to nucleases, pneumococci utilize their polysaccharide capsule and lipoteichoic acid (LTA) modifications to reduce their susceptibility to NETs, demonstrating a multifaceted evasion strategy [33,34].

Importantly, neutrophils tailor their responses based on the pathogen encountered. For larger invaders, such as fungi that resist phagocytosis, NETs become especially vital. Fungal pathogens like Aspergillus are captured and damaged by NET components. Nevertheless, these microbes have also evolved countermeasures, including cell wall polysaccharides and enzymatic degradation of NETs by secreted DNases and nucleotidases [12,35].

Beyond their direct antimicrobial function, NETs also enhance immune responses. Research by Domer et al. shows that NETs can stimulate neutrophils themselves, promoting degranulation, reactive oxygen species (ROS) production by NOX2, and the generation of additional NETs. Moreover, they drive the release of inflammatory mediators like IL-8 and BAFF via intracellular signaling pathways (e.g., Akt, ERK1/2, and p38). These findings reveal that NETs are not merely passive traps but active participants in shaping and amplifying the immune response, which may have implications in both infection control and inflammatory disease pathogenesis [36].

### 3.2. Detrimental Roles of NETs in Sepsis

The inflammatory response during sepsis is dysregulated—though necessary to contain infection, it is exacerbated to levels harmful to the host. Inflammatory cells respond to various danger-associated molecular patterns (DAMPs) and pathogen-associated molecular patterns (PAMPs), both of which amplify inflammation [4]. Neutrophil extracellular traps (NETs) significantly contribute to organ damage in this context, particularly by promoting endothelial dysfunction through multiple mechanisms. NETs are critically implicated in microcirculatory failure through the mechanisms of microvascular occlusion, endothelial injury, and immunothrombosis. Excess NET deposition in the capillaries promotes the production of platelet–neutrophil aggregates, upregulation of tissue factor (TF), disruption of the endothelial glycocalyx, and impaired perfusion [37]. Endothelial sensing of NET-derived DNA activates the cGAS–STING pathway, inducing inflammation and TF expression, thereby amplifying coagulation and exacerbating tissue hypoxia [38]. Here we provide a summary of the role of NETs in macro- and microvascular damage, focusing on endothelial damage and thrombotic processes.

#### 3.2.1. Endothelial Injury and Barrier Disruption

The vascular endothelium is central to the pathophysiology of systemic inflammation. It regulates vascular tone via nitric oxide (NO) production and maintains the barrier’s integrity to prevent leakage of fluids and proteins. Its integrity relies on three main components: the glycocalyx, the extracellular matrix (ECM), and intercellular junctions. The glycocalyx, composed of proteoglycans and glycosaminoglycans (e.g., heparan sulfate, hyaluronic acid, chondroitin sulfate), prevents leukocyte adhesion. The ECM, mainly laminin and collagen IV, contributes to structural support. Intercellular junctions include tight junctions (occludin, claudins, JAMs) and adherens junctions, where VE-cadherin interacts with catenins and the cytoskeleton to ensure the barrier’s integrity [39]. During inflammation, neutrophil–endothelium interaction is required for transendothelial migration (TEM), which involves transient disassembly of adherens junctions. This disassembly is mediated by tyrosine phosphorylation of VE-cadherin and β-catenin. Upon leukocytes binding to ICAM-1, two tyrosine kinases—Src and Pyk2—are activated, leading to phosphorylation of VE-cadherin at Y658 (p120-catenin binding site) and Y731 (β-catenin binding site), facilitating junctional opening for leukocyte transport [40,41]. This mechanism is further amplified by NETs, which increase the endothelial ICAM-1 and VCAM-1 expression while reducing the VE-cadherin levels, resulting in exaggerated vascular permeability. NET-associated endothelial activation and barrier disruption have been implicated in various conditions, including limb ischemia, preeclampsia, and septic encephalopathy [42,43,44]. Notably, citrullinated histone H3, an NET component, directly disrupts adherens junctions and promotes actin cytoskeletal remodeling, further compromising the endothelial barrier [45]. ICAM-1 plays a critical role in this process. Its upregulation during inflammation not only enhances leukocyte adhesion and paracellular migration but also enables transcellular migration. ICAM-1 engagement is a central driver of cytoskeletal rearrangement and adherens junction destabilization, leading to increased permeability and vascular leakage [46,47]. Folco et al. (2018) demonstrated that NETs promote ICAM-1 and VCAM-1 expression in an IL-1α-dependent manner, contributing to endothelial cell activation [48].

Once activated and transmigrated into inflamed tissues, neutrophils can re-enter circulation through a process known as reverse transendothelial migration (rTEM). This phenomenon is only partially understood and appears to be regulated by factors such as macrophage-derived neutrophil redox–Src family kinase signaling and the activity of leukotriene B4 (LTB4). Neutrophils undergoing rTEM exhibit high levels of ICAM-1 expression, a feature associated with their altered migratory behavior. rTEM has been implicated in the systemic propagation of inflammation and the development of remote organ damage [6]. A study in aged murine models revealed that rTEM neutrophils accumulate preferentially at the endothelium of inflamed tissues, primarily driven by CXCL1 release from perivascular mast cells. Once back in circulation, these neutrophils preferentially home to the pulmonary vasculature, where they contribute to endothelial injury. These findings suggest that rTEM neutrophils may mediate secondary, distant tissue damage, beyond that at the original site of inflammation [49].

Endothelial cells themselves, when preactivated by PMA and TNF-α, release IL-8, a potent inducer of NETosis via NADPH oxidase activation. While NETs contribute to infection control in sepsis, their interaction with endothelial cells triggers apoptosis and barrier dysfunction. This is primarily mediated by NET-associated proteases degrading the glycocalyx, exposing adhesion molecules and sustaining neutrophil recruitment in a feed-forward loop [7,50]. An increase in the NET granule contents directly increases the endothelial permeability. NE degrades VE-cadherin in vitro, though its in vivo relevance remains debated. Other neutrophil-derived molecules, such as arachidonic acid metabolites, notably LTB4, further amplify endothelial dysfunction by inducing the production of heparin-binding protein (HBP), which binds endothelial proteoglycans and exacerbates vascular leakage [47]. Moreover, oxidative stress enhances endothelial damage by degrading key glycocalyx components (e.g., SODs, syndecans) using matrix metalloproteinases (MMPs). Elevated histone deacetylase (HDAC) activity under oxidative conditions upregulates MMPs (MMP-2, -8, -9) while downregulating their natural inhibitors (TIMP-1, TIMP-3), contributing to glycocalyx breakdown [51]. Clinical data reveal that MMP-2 and MMP-8 are elevated in patients with severe sepsis, whereas MMP-9 shows a rapid, transient spike, linking to organ damage in septic patients [52].

#### 3.2.2. Sepsis-Induced Thrombosis

An Overview of Coagulation in Sepsis and the Role of NETs: During sepsis, a pathological amplification of the hemostatic response occurs, leading to variable degrees of coagulopathy. Both primary and secondary hemostasis are involved. Platelets become hyperactivated through the influence of bacterial products and circulating mediators, such as IL-6. In this setting, the release of von Willebrand factor (vWF) is increased, enhancing the platelets’ adhesion to the endothelium via GPIIb/IIIa integrins and promoting intravascular microthrombi formation. Concurrently, vascular damage induced by pathogens triggers the tissue factor (TF)-mediated activation of the extrinsic coagulation cascade, primarily involving Factor VII, which can crosstalk with the intrinsic pathway, amplifying the procoagulant response [53].

Disseminated Intravascular Coagulation (DIC): One of the most severe complications of sepsis is disseminated intravascular coagulation (DIC), a condition characterized by widespread microvascular thrombosis and subsequent consumption of clotting factors and platelets. This thrombo-inflammatory state is driven by a dysregulated immune response that leads to an overwhelming release of proinflammatory cytokines and procoagulant mediators, such as TF. The resulting systemic activation of coagulation impairs tissue perfusion and contributes to multi-organ dysfunction [54]. NETs are increasingly recognized as central mediators of this process, bridging innate immunity and thrombogenesis.

NETs and Platelet Aggregation. There is a bidirectional pathological interaction between NETs and platelets. Activated platelets promote NET formation via two main mechanisms: the interaction between P-selectin and its neutrophilic receptor, PSGL-1, and the release of high-mobility group box 1 (HMGB1), a chromatin-binding protein that stabilizes nucleosomes and stimulates neutrophils to undergo NETosis. In return, NETs support platelet recruitment and aggregation by presenting complement component C3b—recognized by platelet CR1 receptors—and by releasing histone H4, a potent activator of platelet aggregation. Histone-induced signaling involves activation of intracellular pathways such as ERK, Akt, p38 MAPK, and NF-κB, promoting the release of vWF and fibrinogen binding [22,55].

NETs and the Extrinsic Coagulation Pathway. Extracellular histones, a key structural component of NETs, have been shown to enhance thrombin generation and inhibit the natural anticoagulant system, particularly by impairing thrombomodulin (TM)-mediated protein C activation. This results in a shift toward a prothrombotic state [56]. Furthermore, histones increase the TF expression and activity in endothelial cells, both at the surface and mRNA levels, while simultaneously suppressing TM expression. This mechanism appears to involve TLR2 signaling, as pharmacological inhibition of TLR2 attenuates histone-induced procoagulant effects [57]. Additionally, NET-associated proteases such as neutrophil elastase and proteinase 3 (PR3) can upregulate TF expression, as demonstrated in ANCA-associated vasculitis models [58]. Another key player is IL-1α, which is activated by cathepsin G released from neutrophils. The mature IL-1α form contained within NETs stimulates endothelial cells, further increasing the TF expression and reinforcing the prothrombotic role of NETs in sepsis [48].

NETs and the Intrinsic Coagulation Pathway. In addition to their influence on the extrinsic pathway, NETs also play a critical role in activating the intrinsic coagulation cascade. One of the most important components involved is cell-free DNA (cfDNA), primarily released by intact NETs. Negatively charged cfDNA has been shown to activate Factor XII, initiating the contact pathway and driving thrombin generation independently of platelets. This mechanism highlights the underestimated role of the intrinsic pathway in septic coagulopathy and may explain why some septic patients do not display overt hypercoagulability if only the extrinsic pathway is considered [59,60]. Furthermore, the cfDNA levels are markedly elevated in septic patients and have been closely correlated with the disease severity and prognosis, sometimes outperforming traditional scoring systems such as APACHE II. The combination of cfDNA measurements with conventional biomarkers may offer improved risk stratification [61,62].

NETs, Clot Stability, and Resistance to Fibrinolysis. Beyond their procoagulant properties, NETs contribute to clot stabilization and resistance to fibrinolysis. The DNA scaffold of NETs not only enhances thrombus formation but also prevents clot dissolution. cfDNA binds both fibrin and plasmin, forming inactive complexes that inhibit plasmin-mediated fibrinolysis. This mechanism contributes to the persistence of intravascular thrombi and the pathogenesis of DIC. Experimental data confirm that thrombi containing NETs are denser, more compact, and significantly more resistant to enzymatic degradation, posing a therapeutic challenge in sepsis-induced thrombosis [59,60].

#### 3.2.3. Pathological Angiogenesis in Sepsis

In addition to their well-established roles in endothelial injury and microvascular thrombosis, emerging evidence indicates that NETs can induce pathological angiogenesis under inflammatory conditions. Aldabbous et al. first demonstrated NET-induced angiogenic activation in human pulmonary artery endothelial cells based on MPO/H_2_O_2_-dependent TLR4/NF-κB signaling, observing increased expression of pro-angiogenic factors like PDGF and heparin-binding EGF-like growth factor in a pulmonary hypertension model [63]. While the direct studies on NET-mediated abnormal angiogenesis in sepsis remain limited, there is a striking mechanistic overlap. During sepsis, NET components—particularly histones and proteases—activate endothelial cells through TLR signaling pathways and NF-κB, leading to upregulation of ICAM-1, VCAM-1, VEGF, and other angiogenic markers. Such endothelial activation occurs within a milieu of cytokine storms and oxidative stress, potentially triggering disorganized neovessel formation rather than physiologic angiogenesis [7]. Moreover, endothelial progenitor cells (EPCs), essential for vascular repair, are quantitatively and functionally altered in sepsis. Elevated EPC counts on presentation, followed by a decline in non-survivors, correlate with worse outcomes, reflecting dysfunctional endothelial regeneration pathways [64]. These observations suggest that NET-induced endothelial damage may impair EPC-mediated neovascular repair and contribute to aberrant angiogenic remodeling. Taken together, it is biologically plausible that NETs contribute not only to immunothrombosis and capillary occlusion but also to pathologic angiogenesis in sepsis, exacerbating microvascular perfusion abnormalities and organ dysfunction. Further research is needed to elucidate these mechanisms in vivo and assess whether therapeutically targeting NETs may mitigate aberrant angiogenesis in sepsis.

## 4. Biomarkers of NET Activation in Inflammation and Sepsis

Several biomarkers associated with NETs have emerged as valuable tools for assessing the disease severity, organ dysfunction, and prognosis in septic patients.

### 4.1. Nucleosomes

Among the most extensively studied are nucleosomes containing histone H3.1, which are closely linked to NETosis and have been identified in multiple studies as both diagnostic and prognostic markers of sepsis. These nucleosomes are elevated from an early stage in septic patients and show a positive correlation with the IL-6 levels and neutrophil counts, highlighting their potential for use as early diagnostic indicators of sepsis. Moreover, elevated H3.1 nucleosome levels are associated with higher disease severity, as measured by the SOFA score, as well as with greater early and 28-day mortality [65]. These levels are particularly high in patients with severe sepsis or septic shock, and they correlate with organ damage, especially DIC, acute kidney injury (AKI), and hyperinflammatory sepsis. Notably, they demonstrate good predictive power for the need for renal replacement therapy [66]. One study identified them as markers of both widespread cell death and neutrophil activation. The citrullinated variant H3R8, in particular, has been shown to correlate with the disease severity in both septic and COVID-19 models, albeit with different expression levels, allowing for differential diagnosis between these conditions. Furthermore, the ratio of H3 to H3R8 provides an estimate of the degree of histone citrullination mediated by PAD4 [67]. Citrullinated H3 histones are early markers of sepsis [68], and in experimental models of sepsis and acute pancreatitis, they are useful indicators of the disease outcomes and severity [69]. Another study investigated H3 nucleosomes as markers of neutrophil activation and NETosis, showing that their levels correlate with the 28-day mortality in patients with sepsis-induced acute kidney injury [70].

### 4.2. MPO and MPO-DNA

MPO, a key enzyme in NET formation, has also been identified in several studies as a marker of neutrophil activation and the disease severity in both sepsis and COVID-19 [67,71]. The MPO-DNA complex, in particular, serves as a potent indicator of systemic inflammation, found at higher levels in septic patients compared to healthy controls, and correlates with the extent of organ damage [72]. A study on patients with Streptococcus bloodstream infections showed significantly higher MPO-DNA levels in those with streptococcal bacteremia, especially in the presence of abscess-forming infections and infective endocarditis, even when co-infected with other highly virulent pathogens. These levels also correlated with increased mortality [73]. MPO-DNA is a recognized marker of NET activation and has been associated with the 28-day mortality in patients with sepsis-induced AKI, similarly to the NE-DNA complex evaluated in the same study [70].

### 4.3. CfDNA

Another key marker of neutrophil activity and the NET composition is cell-free DNA (cfDNA), which is a well-known biomarker of sepsis and organ injury [72]. One study identified cfDNA as being a superior early biomarker of neonatal sepsis when compared to the citrullinated histones and neutrophil elastase (NE) [74]. Circulating DNA, released during thrombotic microangiopathies, exhibits prothrombotic potential—an effect neutralized by DNase treatment—and correlates with the disease activity. Although cfDNA is thought to originate predominantly from NETs, supported by concurrent increases in the MPO and S100 proteins, it may also be derived from tumors or other damaged cells [71].

### 4.4. Nitric Oxide (NO)

Nitric oxide (NO) plays a central role in the pathophysiology of sepsis, with its overproduction by inducible nitric oxide synthase (iNOS) contributing to hypotension, mitochondrial dysfunction, and impaired microvascular perfusion. NO also modulates neutrophil redox signaling and potentially influences NET formation. While studies specifically linking NO levels to NETosis in human sepsis remain limited, combining NO biomarkers with NET-related markers—such as cell-free DNA and MPO-DNA complexes—could enhance prognostic stratification by integrating information on vascular, immune, and coagulative dysfunction. This multi-analyte approach recognizes NO as both a modulator and indicator of endothelial and innate immune dysregulation in septic states [24,75].

### 4.5. Others

Another NET-associated biomarker is high-mobility group box 1 protein (HMGB1), particularly its lactylated form, which has been implicated in lactate-induced AKI, thereby linking NET activity to kidney injury [76]. Gene expression profiling has identified several NET-related genes—ELANE, TLR4, MPO, PADI4, CTSG, MMP9, and S100A12—as being potential diagnostic biomarkers and therapeutic targets for sepsis [77]. Another study pinpointed that two key genes involved in NETosis, CYBB and FCAR, were independent predictors of poor survival and promising targets for mitigating NET-mediated lung damage [78]. The heterodimer S100A8/A9 (calprotectin) is another NET-associated molecule, released during neutrophil activation and potentially during NETosis, that reflects the disease activity in thrombotic microangiopathies [71]. The balance between NET formation and degradation is further regulated by DNases, especially DNase1L3, whose reduced expression in severe cases impairs the NET clearance. A reduction in plasmacytoid dendritic cells—the primary source of DNase1L3—can exacerbate this imbalance [79]. Finally, N-formyl methionine peptides (fMET) of mitochondrial origin, recognized as damage-associated molecular patterns (DAMPs), enhance neutrophil activation and NET release. Elevated levels of these peptides have been observed in critically ill COVID-19 patients, emphasizing the contribution of mitochondrial signals to NET-driven inflammation [80] (Table 1).

## 5. Therapeutic Strategies Targeting NETs

### 5.1. Inhibition of NETosis

#### 5.1.1. PAD4

PAD4 is a neutrophil enzyme involved in the conversion of arginine to citrulline, a critical step in the formation of NETs. Excessive histone citrullination, already recognized as a risk factor for autoimmune diseases, suggests that targeting PAD4 with specific inhibitors could be beneficial in conditions where NETs play a pathogenic role [81]. The use of anti-PAD4 molecules—such as Calcium, rTcpC, JBI-589, GSK199, GSK484, and TDFA—has been proposed as a means to reduce NETosis, which in turn could mitigate the clinical impact of one of the most severe complications of sepsis: acute respiratory distress syndrome (ARDS) [82]. Some researchers have developed a system utilizing collagen IV-targeted nanoparticles (Col IV NPs) to deliver the PAD4 inhibitor GSK484 specifically to the damaged vascular endothelium, where type IV collagen in the basement membrane is exposed. This targeted approach aims to reduce NETosis-induced endothelial injury [83]. The same inhibitor, GSK484, has also demonstrated protective effects on the blood–brain barrier in models of sepsis-associated encephalopathy by reducing neuroinflammation through the inhibition of NET formation [44]. Additionally, GSK484-mediated NETosis inhibition has been studied in a cohort of patients with type 2 diabetes, where it led to reduced neutrophil infiltration in the gut and amelioration of intestinal barrier dysfunction [84]. In neonatal models of infectious peritonitis, another PAD4 inhibitor, Cl-amidine, decreased the peritoneal NET infiltration and inflammatory cytokine levels, resulting in improved survival compared to the controls [85]. In murine models of sepsis, treatment with Forsythiaside B was shown to downregulate PAD4 expression and reduce NET formation, with beneficial effects on sepsis-associated coagulopathy [86]. As previously mentioned, the investigation of PAD4 inhibitors extends beyond sepsis and infectious diseases, showing promising results in other conditions such as rheumatoid arthritis [87], Kawasaki disease [88], ulcerative colitis [89], and cancers [90].

#### 5.1.2. ROS

The presence of ROS is essential for suicidal NETosis; therefore, reducing the ROS levels may attenuate the impact of NETs in septic processes. Among the most extensively studied antioxidant molecules are vitamins C and E, as well as N-acetylcysteine. These agents, by lowering the ROS levels, exert an inhibitory effect on NET formation and have been associated with beneficial outcomes in terms of systemic inflammation and tissue damage [91,92]. One study demonstrated that hesperetin (HES), a flavonoid with potent antioxidant properties, reduces ROS production and subsequently inhibits NET formation in vitro. In vivo, in murine models of sepsis, HES decreased the cytokine levels and organ dysfunction [93]. Another antioxidant with anti-inflammatory properties, zingerone, also significantly reduced NETosis and systemic inflammation in animal models of sepsis through ROS inhibition [94]. An in vitro study on human neutrophils investigated the antioxidant role of molecular hydrogen (H_2_), showing that it decreases ROS production and impacts neutrophil aggregation, histone citrullination, and the release of NET components [95]. A positive effect was also observed in vitro with another antioxidant and anti-inflammatory compound, octyl gallate (OG). OG acts as an ROS inhibitor and consequently suppresses NET formation, although these findings have yet to be confirmed in animal models [96]. Interestingly, even antibiotic de-escalation in sepsis has been shown to reduce NET-mediated organ damage in a ROS-dependent manner [97].

#### 5.1.3. MPO

MPO plays a critical role in NETosis, prompting some studies to investigate the use of MPO inhibitors in NET-associated diseases. A study conducted in murine models of myocarditis demonstrated that the MPO inhibitor PF-1355 attenuates myocardial injury by modulating and reducing NETosis, thereby limiting the NET-related pyroptotic damage [98]. Another inhibitor, AZD5904, has been employed as an experimental tool to confirm that MPO activity is directly responsible for oxidative modifications within NETs. These findings support the hypothesis that MPO inhibition could represent a promising therapeutic strategy to mitigate the pathological effects of NETs in various inflammatory conditions [99].

### 5.2. Degradation of Already Formed NETs

#### DNASe

DNase I is arguably the most extensively studied molecule targeting NETs and has already been safely used in clinical settings. Unlike agents that prevent NETs’ formation, DNase I does not interfere with their generation but rather alters their structure by degrading cfDNA, a key structural component of NETs. This mechanism has shown promising results in neoplastic, inflammatory, and septic conditions. However, some studies have reported that DNase I treatment does not consistently reduce the inflammatory cytokine levels, likely because it does not act on other equally pathogenic NET components such as histones and proteolytic enzymes [100]. The beneficial effects of DNase I appear to result from reduced neutrophil infiltration and NET accumulation in affected tissues, ultimately providing protection against sepsis-associated organ damage. Indeed, animal models of sepsis have demonstrated that DNase I administration can prevent lung injury and improve the survival in cases of ARDS, without affecting the bacterial load and acting specifically on NET degradation. This highlights DNase I as a potential adjuvant therapy to antibiotics in the management of septic patients [101]. A similar protective mechanism was observed in a study using animal models of transfusion-related acute lung injury (TRALI), where inhaled DNase I reduced the alveolar NET concentrations and improved the clinical outcomes, including oxygen saturation [102]. Additionally, DNase I may help prevent sepsis-associated immunothrombosis, although further studies are needed to confirm this role [103].

DNase I has also been shown to reduce blood–brain barrier disruption and neuronal apoptosis, thereby preventing sepsis-associated encephalopathy, by targeting neutrophils and NETs [104]. Interestingly, septic patients often exhibit reduced endogenous DNase I levels, further supporting the rationale for its therapeutic supplementation in this population [105]. A similar mechanism has been implicated in the increased mortality observed in patients with severe COVID-19 pneumonia, which has been linked to impaired DNA clearance due to suppressed endogenous DNase activity. These findings reinforce the potential utility of therapeutic strategies aimed at enhancing NET degradation [106]. Importantly, the therapeutic potential of DNase I extends beyond sepsis. For instance, in a murine myocardial infarction model, co-administration of unfractionated heparin and DNase I was found to reduce the NET infiltration in cardiac tissue and significantly limit myocardial injury [107].

### 5.3. Blocking the Pathogenic Effects of NETs

#### 5.3.1. Heparin

Heparin, widely known in clinical practice for its anticoagulant properties, also exerts beneficial effects on NETs-related complications that are independent of its anticoagulant activity. Due to its negative charge, heparin can bind positively charged histones, which are known to contribute to endothelial damage and thrombosis [108]. In one study, heparin immobilized on adsorbent surfaces demonstrated an effective capacity to bind and remove key components contributing to immunothrombosis, including NETs, histones, PF4, and HMGB1, suggesting a potential therapeutic role in mitigating the systemic thrombo-inflammatory response [109]. In a cohort of patients with COVID-19, heparin administration was associated with a dose-dependent reduction in the NETosis levels and a decrease in major NET-associated biomarkers, highlighting its potential as a therapeutic agent [110]. Furthermore, heparin appears to enhance DNase I-mediated degradation of DNA–histone complexes [105] and promote NE-mediated histone proteolysis, thereby reducing the cytotoxic potential of extracellular histones [111].

#### 5.3.2. Aspirin

We also mention Acetyl Salicylic Acid (ASA), a well-known anti-platelet and anti-inflammatory molecule with potential therapeutic applications in the field of NETosis. ASA directly suppresses NETosis in human neutrophils by inhibiting NF-κB p65 phosphorylation: treatment with ASA abolished PMA- and TNF-α-induced NET formation, an effect recapitulated by the NF-κB inhibitors BAY 11-7082 and Ro 106-9920 and confirmed in a mouse peritonitis model where ASA pretreatment markedly decreased the NET content [112]. Furthermore, ASA-treated platelets exhibit reduced release of platelet factor 4 (PF4) and von Willebrand factor (vWF), limiting platelet-driven NETosis under an LPS or arachidonic acid challenge [22]. Collectively, these data position aspirin as a promising anti-NET therapeutic agent, although clinical translation must carefully balance its antithrombotic efficacy against the risk of bleeding complications.

### 5.4. Novel Approaches

Some molecules are emerging as central targets in the search for new therapeutic strategies. A recent study showed that plasma from septic patients induces significantly higher NET formation compared to plasma from patients with non-septic systemic inflammation and that this NETosis correlates with the circulating levels of CXCR1/2 chemokines, such as IL-8. In murine models of sepsis, pharmacological inhibition of CXCR1/2 with reparixin markedly reduced the NET formation, multi-organ damage, and mortality, without impairing bacterial clearance [113]. Another study demonstrates that the circadian factor BMAL1 regulates innate immunity in sepsis and protects against sepsis-induced ALI by suppressing CXCL2 expression. Its deficiency in macrophages promotes neutrophil recruitment and NET formation via the CXCR2 receptor, worsening inflammation and organ damage, while inhibition of CXCR2 with SB225002 significantly reduces neutrophil infiltration, NETosis, and multi-organ dysfunction, suggesting a potential therapeutic target [114]. In abdominal sepsis models, the NET formation and intestinal infiltration are increased and correlate with enterocyte injury. In PAD4-deficient mice, the levels of an enzyme essential for NET formation, intestinal barrier damage, and endoplasmic reticulum (ER) stress are reduced, while in vitro NETs induce epithelial barrier disruption and ER stress. Inhibition of the TLR9 receptor decreases NET-mediated intestinal epithelial cell death, suggesting that the TLR9-ER stress pathway is a potential therapeutic target to mitigate NET-induced intestinal dysfunction in sepsis [115].

### 5.5. Extracorporeal Sorption Strategies for NET Clearance

The removal of circulating NET components via extracorporeal sorption has emerged as a promising adjunctive strategy in septic patients. Hemoperfusion with polymyxin B-immobilized cartridges (PMX-HP) has been shown, both ex vivo and in vivo, to reduce the plasma levels of NET-associated biomarkers—MPO-DNA, NE-DNA, and cell-free DNA—in subjects with septic shock, supporting the concept of selective NET sorption [116]. More generally, extracorporeal blood purification techniques—including the use of adsorption cartridges and high-cut-off membranes—demonstrate the ability to remove inflammatory mediators and DAMPs, which likely extends to NET-associated molecules, although clinical trials specifically targeting NET clearance remain pending. Several examples can be given. Polymyxin B columns selectively bind endotoxin in Gram-negative sepsis but show mixed clinical results, benefiting only patients with moderate endotoxin levels. The LPS Adsorber uses peptides to capture endotoxin, though the clinical evidence for this is limited. CytoSorb^®^ (CytoSorbents Corporation, Princeton, NJ, USA) non-selectively adsorbs cytokines and other mediators, with promising effects on the hemodynamics but insufficient proof of a mortality benefit. oXiris^®^ (Baxter, Pune, Maharashtra) is a special filter that removes inflammation-causing substances and toxins in septic shock, showing promising but not yet conclusive results. CPFA filters plasma to clear toxins but has not proven beneficial in trials and is unlikely to be used for sepsis. Albumin dialysis removes large, albumin-bound toxins mainly found in liver failure patients; its role in sepsis treatment remains unclear, and it is costly. Overall, these techniques hold potential, but the optimal patient selection criteria and timing are still being defined. These approaches may reduce endothelial injury, coagulation activation, and microvascular occlusion by lowering the systemic burden of NETs [117] (Table 2).

## 6. Conclusions

Neutrophil extracellular traps (NETs) play a central role in the immune response to sepsis, acting as both antimicrobial agents and key drivers of inflammation, thrombosis, and organ injury. While their formation represents an essential component of host defense, excessive or dysregulated NETosis contributes significantly to the disease severity and mortality in septic patients. The identification of NET-associated biomarkers—such as circulating nucleosomes (including histone H3.1 and citrullinated H3), myeloperoxidase–DNA (MPO-DNA) and neutrophil elastase–DNA (NE-DNA) complexes, cell-free DNA, and NET-related gene expression profiles—has advanced our understanding of NETs as both diagnostic and prognostic tools. These markers correlate with organ dysfunction, coagulation abnormalities, and poor outcomes and may help distinguish septic patients from those with other inflammatory conditions like COVID-19. An imbalance between NET formation and degradation, particularly due to reduced DNase activity and impaired function of plasmacytoid dendritic cells, exacerbates the pathological effects of NETs. This insight has opened new avenues for therapeutic intervention. Experimental strategies targeting NETs include the use of DNase enzymes to promote NET degradation, PAD4 inhibitors to prevent histone citrullination, and agents that modulate neutrophil activation pathways such as MPO or NE inhibitors. Additionally, recent work has identified NET-related genes (e.g., CYBB, FCAR, PADI4) as potential therapeutic targets. Incorporating NET biomarkers into clinical practice could improve early diagnosis, risk stratification, and monitoring of the therapeutic response in septic patients. However, translating these findings into effective, targeted therapies requires further validation through clinical trials. A better understanding of the temporal and mechanistic dynamics of NETosis in sepsis will be key to developing personalized, NET-focused therapeutic strategies.

## 7. Methods

The search was performed between April and May 2025 in the PubMed database. English-language original papers, short communications, clinical trials, randomized controlled trials, meta-analyses, letters, editorials, and articles were evaluated. Emphasis was placed on the selection of original papers and randomized controlled trials, which were filtered to exclude articles not published in the last five years, whenever possible.

## Figures and Tables

**Table 1:** Biomarkers of NET activation in sepsis and inflammation.

Biomarker	Source/Mechanism	Clinical Relevance	Associated Conditions
Histone H3.1 nucleosomes	Released during cell death and neutrophil activation	Correlates with DIC, AKI, hyperinflammation, and 28-day mortality	Sepsis, COVID-19
Citrullinated H3 (e.g., H3R8)	NET-specific modification by PAD4	Indicates NET activation; associated with disease severity and prognosis; elevated in early sepsis	Sepsis, acute pancreatitis, COVID-19
HMGB1 (lactylated)	DAMP, promotes release of NETs in response to lactate	Induces NETosis; contributes to AKI development	Sepsis, lactate-induced AKI
NET-related genes	ELANE, TLR4, MPO, PADI4, CTSG, MMP9, S100A12, CYBB, FCAR	Diagnostic and prognostic value; potential therapeutic targets; predict poor survival; targeting these reduces NET formation and lung injury	Sepsis
MPO	Neutrophil granule enzyme involved in ROS-dependent NETosis	Marker of NET activation and disease severity; part of MPO-DNA complexes	Sepsis, COVID-19, TMA
MPO-DNA complex	Marker of extracellular NET release	Correlates with inflammation, coagulation, organ damage, and bacteremia mortality	Sepsis, infective endocarditis
NE-DNA complex	Derived from neutrophil elastase during NETosis	Indicator of NET activation; correlates with AKI and 28-day mortality	Sepsis with AKI
S100A8/A9 (calprotectin)	Neutrophil-derived protein complex	Reflects neutrophil activation and NETosis; increases with disease activity	Thrombotic microangiopathies, sepsis
Cell-free DNA (cfDNA)	Released from NETs or damaged cells	Marker of inflammation and organ damage; effective in early sepsis detection, including in neonates	Sepsis, neonatal sepsis
DNases (e.g., DNase1L3)	Enzymes degrading extracellular DNA	Impaired degradation in severe disease; therapeutic interest; linked to pDC dysfunction	Severe sepsis, COVID-19
fMET peptides	Mitochondrially derived DAMPs	Activate neutrophils and trigger NETosis; elevated in severe cases	COVID-19

**Table 2:** Summary of therapeutic strategies addressing NETosis.

Therapeutic Strategy	Molecular Target	Mechanism of Action	Examples	Indications/Notes
PAD4 inhibitors	Peptidyl arginine deiminase 4 (PAD4)	Block histone citrullination → prevent DNA decondensation	Cl-amidine, GSK484	Autoimmune diseases, sepsis
ROS inhibitors	Reactive oxygen species (ROS)	Reduce oxidative stress necessary for NETosis	N-acetylcysteine, apocynin	Chronic inflammation, sepsis
MPO inhibitors	Myeloperoxidase (MPO)	Inhibit enzyme involved in NETosis	PF-1355, AZD5904	Autoimmune and cardiovascular diseases
DNases	Extracellular DNA	Degrades DNA backbone of NETs	DNase I (dornase alfa)	Cystic fibrosis, sepsis, ARDS, COVID-19
Histone neutralization	Histones	Neutralizes extracellular histone toxicity	Heparin, anti-histone antibodies	Reduces endothelial damage and thrombosis
Heparin and derivatives	Histones, clots	Anticoagulants and neutralize NETs	Heparin, low-molecular-weight heparins	Venous thrombosis, severe COVID-19
Monoclonal antibodies	Specific NET components or receptors (e.g., TLR9)	Block NET activation or effects	Under investigation	Various preclinical models
Upstream signaling inhibitors	Cytokines and receptors (IL-8, CXCR2, TLR)	Reduce NETosis induction	Experimental molecules	New therapeutic perspectives
oXiris^®^ hemofilter, (Baxter, Pune, Maharashtra)	Inflammatory mediators, endotoxin, uremic toxins	Adsorbs cytokines and endotoxins; removes fluids and toxins	oXiris^®^ filter (AN69 membrane) (Baxter, Pune, Maharashtra)	Sepsis, septic shock with renal failure; preliminary positive results, further RCTs needed
Cytosorb^®^ (CytoSorbents Corporation, Princeton, NJ, USA)	Cytokines, inflammatory mediators	Adsorbs inflammatory mediators and toxins using polymer resin	Cytosorb^®^ (CytoSorbents Corporation, Princeton, NJ, USA)	Sepsis, systemic inflammation; used as extracorporeal supportive therapy
Coupled plasma filtration adsorption (CPFA)	Plasma and inflammatory mediators	Plasma separated and purified by resin adsorption, then reinfused	CPFA	Sepsis; limited evidence, negative RCT results, no longer recommended
Albumin dialysis MARS^®^ (Vantive Health LLC, Deerfield, IL, USA), Prometheus^®^ (Fresenius Medical Care, Bad Homburg, Germany), ADVOS^®^ (ADVITOS GmbH, Munich, Germany)	Albumin-bound toxins and inflammatory mediators	Removes albumin-bound toxins using albumin-containing dialysate	MARS^®^ (Vantive Health LLC, Deerfield, IL, USA), Prometheus^®^ (Fresenius Medical Care, Bad Homburg, Germany), ADVOS^®^ (ADVITOS GmbH, Munich, Germany)	Extracorporeal liver support; high cost, complex; role in sepsis unclear
